# Maternal Transmission of Human OGG1 Protects Mice Against Genetically- and Diet-Induced Obesity Through Increased Tissue Mitochondrial Content

**DOI:** 10.3389/fcell.2021.718962

**Published:** 2021-09-15

**Authors:** Natalie Burchat, Priyanka Sharma, Hong Ye, Sai Santosh Babu Komakula, Agnieszka Dobrzyn, Vladimir Vartanian, R. Stephen Lloyd, Harini Sampath

**Affiliations:** ^1^Rutgers Center for Lipid Research, New Jersey Institute for Food, Nutrition, and Health, Rutgers University, New Brunswick, NJ, United States; ^2^Laboratory of Cell Signaling and Metabolic Disorders, Nencki Institute of Experimental Biology, Warsaw, Poland; ^3^Oregon Institute of Occupational Health Sciences, Oregon Health and Science University, Portland, OR, United States; ^4^Department of Molecular and Medical Genetics, Oregon Health and Science University, Portland, OR, United States; ^5^Department of Nutritional Sciences, Rutgers University, New Brunswick, NJ, United States; ^6^Center for Microbiome, Nutrition, and Health, New Jersey Institute for Food, Nutrition, and Health, Rutgers University, New Brunswick, NJ, United States

**Keywords:** DNA repair, metabolic syndrome (in offspring), obesity, developmental origins of disease, mitochondrial function

## Abstract

Obesity and related metabolic disorders are pressing public health concerns, raising the risk for a multitude of chronic diseases. Obesity is multi-factorial disease, with both diet and lifestyle, as well as genetic and developmental factors leading to alterations in energy balance. In this regard, a novel role for DNA repair glycosylases in modulating risk for obesity has been previously established. Global deletion of either of two different glycosylases with varying substrate specificities, Nei-like endonuclease 1 (NEIL1) or 8-oxoguanine DNA glycosylase-1 (OGG1), both predispose mice to diet-induced obesity (DIO). Conversely, enhanced expression of the human *OGG1* gene renders mice resistant to obesity and adiposity. This resistance to DIO is mediated through increases in whole body energy expenditure and increased respiration in adipose tissue. Here, we report that *hOGG1* expression also confers resistance to genetically-induced obesity. While Agouti obese (*A^y^/a*) mice are hyperphagic and consequently develop obesity on a chow diet, *hOGG1* expression in *A^y^/a* mice (*A^y^/a^Tg^*) prevents increased body weight, without reducing food intake. Instead, obesity resistance in *A^y^/a^Tg^* mice is accompanied by increased whole body energy expenditure and tissue mitochondrial content. We also report for the first time that OGG1-mediated obesity resistance in both the *A^y^/a* model and DIO model requires maternal transmission of the *hOGG1* transgene. Maternal, but not paternal, transmission of the *hOGG1* transgene is associated with obesity resistance and increased mitochondrial content in adipose tissue. These data demonstrate a critical role for OGG1 in modulating energy balance through changes in adipose tissue function. They also demonstrate the importance of OGG1 in modulating developmental programming of mitochondrial content and quality, thereby determining metabolic outcomes in offspring.

## Introduction

Oxidatively-induced damage to both nuclear and mitochondrial DNA is repaired *via* the base-excision repair (BER) pathway, initiated by DNA glycosylases. The most commonly formed oxidative lesion, 8-oxoguanine (8-oxoG), is recognized and cleaved by the enzyme, 8-oxoG DNA glycosylase (OGG1). OGG1 initiates repair of 8-oxoG in both the nuclear and mitochondrial genome and has been shown to play a role in diverse pathologies, including neurodegenerative disease, various cancers, and metabolic dysfunction (Sampath et al., 2012a; Vartanian et al., 2017; Sampath and Lloyd, 2019). We have previously demonstrated that mice lacking endogenous OGG1 (*Ogg1*^–^*^/^*^–^) are prone to diet-induced obesity (DIO) and its sequelae, including insulin resistance, ectopic lipid accumulation in liver and skeletal muscle, gut dysbiosis, and chronic inflammation (Sampath et al., 2012b; Vartanian et al., 2017; Simon et al., 2020). Conversely, enhanced expression of human *OGG1* downstream of a constitutive mitochondrial targeting sequence protects mice from DIO, insulin resistance, and adipose tissue inflammation (Komakula et al., 2018). This metabolic protection in *hOGG1* transgenic mice (*Ogg1*^*Tg*^) was accompanied by increases in whole body energy expenditure and increased mitochondrial content and respiration in white adipose tissue (WAT) (Komakula et al., 2018). Thus, our previous studies reported this novel role for OGG1 in modulating energy balance in the context of a hypercaloric high-fat diet (HFD). In the current study, we sought to explore whether *hOGG1* expression could confer protection against genetically-induced obesity. Several models of genetically-induced obesity become obese due to chronic hyperphagia (Robinson et al., 2000; Ellacott and Cone, 2006; Chang et al., 2018). In most of these models, obesity develops spontaneously on a chow diet, without requiring the use of a hypercaloric HFD. We therefore asked the question of whether *hOGG1* expression would be protective in the context of genetically-induced obesity. To address this question, we transferred the gene expressing human *OGG1* downstream of a constitutive mitochondrial targeting sequence (*Ogg1*^*Tg*^) (Wang et al., 2011; Komakula et al., 2018) into the *A^y^/a* obese mouse (*A^*y*^/a)*. The *A^y^/a* model develops hyperphagia and consequent obesity and insulin resistance due to ectopic overexpression of the Agouti protein, an antagonist of the melanocortin receptor (Klebig et al., 1995; Moussa and Claycombe, 1999; Tschöp and Heiman, 2001). Interestingly, our studies indicate an important function for OGG1 in protecting against genetically-induced obesity. Further, they also uncover a critical role for maternal OGG1 genotype in determining obesity resistance both in the context of genetically-induced as well as DIO.

## Experimental Approach

### Animals

Age-matched mice on the C57BL6J background were used in all studies. The generation of *hOGG1*-expressing mice (*Ogg1*^*Tg*^) has been previously described (Wang et al., 2011). *A^y^/a* mice were obtained from Jackson Labs (stock #000021) (Siracusa et al., 1987; Klebig et al., 1995). *A^y^/a* mice expressing the *hOGG1* transgene are designated as *A^y^/a^Tg^* and were bred in house by mating *A^y^/a* or *A^y^/a^Tg^* animals with *Ogg1*^*Tg*^ animals. Cohort sizes were as follows: males– *A^y^/a*: 10; *A^y^/a^Tg^*: 11; females – *A^y^/a*: 10; *A^y^/a^Tg^* 11; *A^y^/a^Tg^*^–^^*dad*^: 5; all HFD studies—*n* = 5–9 males per cohort. For studies using *A^y^/a* mice, body weights were measured weekly from week 4 onward, and food intake was measured weekly between weeks 10–22 for males and 18–22 for females. At 50 weeks of age, body composition was measured by NMR (Echo Medical Systems, Houston, TX, United States) and energy expenditure and physical activity were measured *via* open circuit indirect calorimetry (CLAMS Comprehensive Lab Animal Monitoring System, Columbus Instruments, Columbus, OH, United States). Following an acclimation period of 24 h, oxygen consumption (VO_2_) and carbon dioxide production (VCO_2_) were recorded every minute for 48 h, with a room air reference taken following each cycle of measurements. Beam breaks on the X, Y, and Z axes were tallied for voluntary physical activity measurements. Gonadal WAT, subscapular brown adipose tissue (BAT), liver, and gastrocnemius were collected at 52 weeks of age. For HFD feeding studies, 8-week old male mice were individually housed and given *ad libitum* access to a 60% HFD for 12 weeks, and body weights and food intake were measured weekly. Body composition was assessed, and tissues were collected at 20 weeks of age. All mice were euthanized between 9 and 11 am by isoflurane overdose followed by exsanguination *via* cardiac puncture. For all *in vivo* procedures, every effort was made to minimize discomfort and suffering, in accordance with the protocols approved by the Animal Care and Use Committee of Rutgers University, New Brunswick, New Jersey under protocol No. 201900077.

### Hepatic Lipids

Hepatic lipids were extracted and separated by thin-layer chromatography, as we have previously described (Komakula et al., 2018).

### DNA and RNA Analyses

RNA was isolated using QIAzol Lysis Reagent and the Qiagen RNeasy kit. Superscript III first-strand synthesis system (Invitrogen, Carlsbad, CA, United States) was used to synthesize cDNA from 1 μg of RNA. Quantitative real-time PCR (qPCR) was performed on a QuantStudio 3 Real-Time PCR System (Applied Biosystems, Foster City, CA, United States) with gene-specific primers. Data were normalized to the expression of RNA18SN5 and quantification was done using the 2^–ΔΔ*Ct*^ method. Relative copy number was quantified by qRT-PCR amplification of hOGG1 from 10 ng genomic DNA and normalized to expression of GAPDH.

### Protein Analyses

Whole cell lysates were prepared from frozen tissue using HEPES homogenization buffer (50 mM pH7.4 HEPES, 150 mM NaCl, 10 mM Na-pyrophosphate, 2 mM EDTA, 1% NP-40, 10% Glycerol) with EDTA-free protease and phosphatase inhibitors. Samples are representative of 3–7 animals, and lysate protein concentrations were determined *via* Bradford assay. Equal amounts of protein were separated by SDS-PAGE and transferred to nitrocellulose membranes. Ponceau total protein staining was performed to confirm uniform loading and transfer of proteins. Following this, membranes were blocked with 5% non-fat dried milk in Tris-buffered saline (TBS, 150 mM NaCl, 50 mM Tris–HCl, pH 7.4) with 0.1% Tween 20 at room temperature with shaking for an hour. The membranes were then incubated overnight at 4°C with primary antibody. Primary antibodies used were as follows: VDAC (Pierce Biotechnology, Waltham, MA, United States), COXIV (Abcam, Cambridge, United Kingdom), PGC-1α (Novus Biologicals, Littleton, CO, United States), HSP60, SIRT1, and GAPDH (all from Cell Signaling Technology, Danvers, MA, United States). Membranes were incubated with HRP- or Alexa-fluor conjugated secondary antibodies and signal was detected using enhanced chemiluminescence or fluorescence imaging, respectively, on an Azure c600 imaging system (Azure Biosystems, United States).

### Statistical Analyses

Data are expressed as mean ± SEM for biological replicates; statistical comparisons were carried out by student’s *t*-test for 2-group comparisons and by two−way ANOVA for multi−group comparisons, followed by post−hoc analysis (Bonferroni) in Graph Pad Prism (version 8.2.0 for Windows, GraphPad Software, La Jolla, CA, United States).

## Results

### *hOGG1* Expression Attenuates Body Weight in *A^y^/a* Obese Mice

**FIGURE 1:**
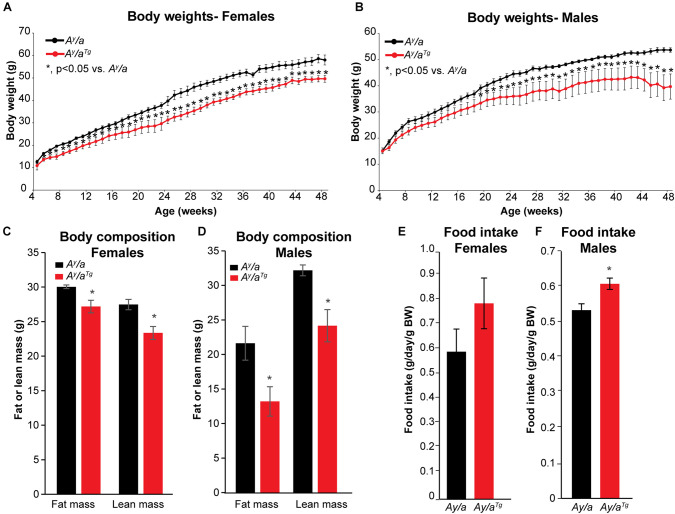
Body weights, body composition, and food intake. **(A,B)** Body weights were reduced in *A^y^/a^Tg^* mice, relative to *A^y^/a* obese counterparts. **(C,D)** Reductions in both lean and fat mass were apparent in *A^y^/a^Tg^* mice. **(E,F)** Food intake was measured weekly between weeks 10–22 for males and 18–22 for females; food intake was slightly increased in *A^y^/a^Tg^* mice (NS in females). Averages ± SEM. *p < 0.05 vs. sex-matched *A^*y*^/a.*

*A^y^/a* yellow obese mice are a genetic model of obesity resulting from antagonism of the melanocortin receptor and consequent hyperphagia (Moussa and Claycombe, 1999; Tschöp and Heiman, 2001). Unlike other common models of obesity such as the leptin-deficient ob/ob or leptin-receptor deficient db/db mice, *A^y^/a* mice express both a functional leptin gene and a leptin receptor. However, these mice develop insulin and leptin resistance, and are hence a valuable model to study genetically-induced obesity in a clinically relevant manner (Klebig et al., 1995; Miltenberger et al., 1997; Tschöp and Heiman, 2001; Rahmouni et al., 2002; Miyazaki et al., 2009). Melanocortin receptor antagonism in *A^y^/a* mice results in chronic hyperphagia and consequent obesity, which is apparent starting at about 16 weeks on a standard rodent chow diet. To determine the potential impact of *hOGG1* expression on body weight in this model, we introduced the *hOGG1* transgene into *A^y^/a* mice to generate *A^y^/a*; *Ogg1*^*Tg*^ (*A^y^/a^Tg^*) mice. Body weights were measured from weaning until 48 weeks of age on a standard chow diet in male and female *A^y^/a* a*nd A^*y*^/a^*Tg*^* littermates. Female *A^y^/a^Tg^* mice had significantly lower body weights than *A^y^/a* counterparts, starting as early as 5 weeks of age. Male *A^y^/a^Tg^* mice also had significantly lower body weights than their *A^y^/a* counterparts, starting at 21 weeks of age (Figures 1A,B).

Unlike in DIO, which is characterized by increases in fat mass, genetically-induced obesity in *A^y^/a* mice results in increases in both lean and fat masses (Miltenberger et al., 1997). Body composition analyses at 52 weeks of age indicated that reductions in body weight in *A^y^/a^Tg^* mice were reflected in decreases in both lean and fat masses (Figures 1C,D). Thus, *hOGG1* expression in this model attenuates aberrant increases in both lean and fat compartments of *A^y^/a* mice. Increased body weight in *A^y^/a* mice is a consequence of chronic hyperphagia. Interestingly, both female and male *A^y^/a^Tg^* had higher food intakes than their *A^y^/a* counterparts (Figures 1E,F). This suggested that rather than due to attenuation of food intake, peripheral mechanisms may mediate the observed reductions in body weight in *A^y^/a^Tg^* mice.

### *hOGG1* Expression Increases Energy Expenditure and Mitochondrial Content in *A^y^/a* Obese Mice

**FIGURE 2:**
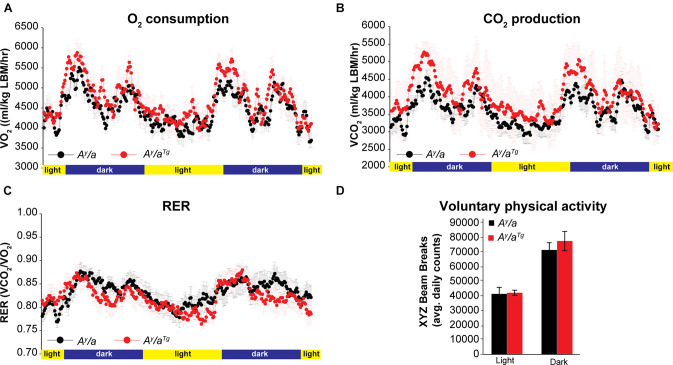
Energy expenditure. **(A)** O_2_ consumption and **(B)** CO_2_ respiration were measured by indirect calorimetry in female mice. **(C)** Respiratory exchange ratios (RER) were calculated during indirect calorimetry measurements. **(D)** Voluntary physical activity was measured during indirect calorimetry. *n* = 6. Averages ± SEM.

Given the similarities between male and female animals, we focused our downstream studies on female mice due to their larger sample size. Energy expenditure analyses were carried out using open circuit indirect calorimetry. *A^y^/a^Tg^* mice had a trend for elevated O_2_ consumption and CO_2_ respiration (Figures 2A,B); however, this was not statistically significant. These data suggested that the reduction in body weights in *A^y^/a^Tg^* mice may be due to physiologically relevant elevations in energy expenditure. Respiratory exchange ratios (RER) were unchanged by genotype (Figure 2C), indicating no differences in whole body substrate utilization. Physical activity was not significantly different between genotypes (Figure 2D).

**FIGURE 3:**
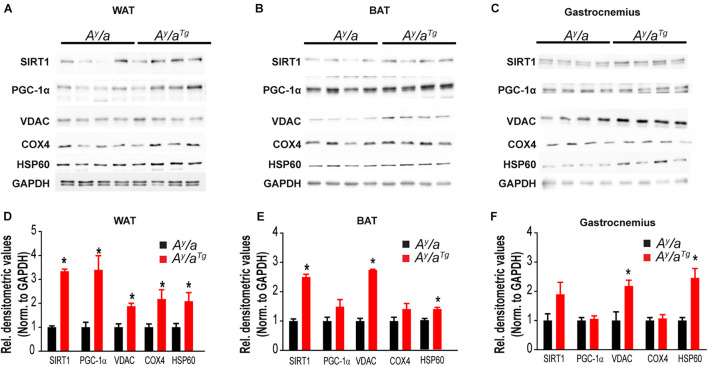
Tissue mitochondrial content. Tissue expression of mitochondrial markers was measured by immunoblotting in **(A,D)** WAT, **(B,E)** BAT, and **(C,F)** Gastrocnemius. BAT, brown adipose tissue, COX4, cytochrome c oxidase; GAPDH, glyceraldehyde 3-phosphate dehydrogenase; HSP60, heat shock protein-60; PGC-1α, peroxisome proliferator-activated receptor gamma coactivator 1-alpha; SIRT1, Sirtuin 1; VDAC, voltage-dependent anion channel; WAT, white adipose tissue.

To investigate metabolic changes that contribute to this lean phenotype and elevated energy expenditure, we evaluated markers of mitochondrial content and energy sensing across multiple metabolically active tissues in *A^y^/a* and *A^y^/a^Tg^* mice (Figure 3). Markers of mitochondrial content, including heat-shock protein 60 (HSP60), voltage-dependent anion channel (VDAC), and cytochrome c oxidase IV (COX4) were significantly elevated in WAT of *A^y^/a^Tg^* mice, relative to *A^y^/a* counterparts. HSP60 and VDAC were increased in BAT and gastrocnemius of *A^y^/a^Tg^* mice (Figures 3A–F). In addition, expression of peroxisome proliferator-activated receptor gamma coactivator-1-alpha (PGC-1α), a master regulator of mitochondrial biogenesis, was elevated across tissues in *A^y^/a^Tg^* mice (NS in gastrocnemius) (Figures 3A–F). Expression of the deacetylase, Sirtuin 1 (SIRT1), which activates PGC-1α, was also significantly increased in WAT of *A^y^/a^Tg^* mice. The elevation in SIRT1 and PGC-1α are consistent with the observed increases in mitochondrial content in these animals. They are also consistent with prior reports from our lab and others indicating a role for OGG1 in altering tissue mitochondrial content and function (Rachek et al., 2002, 2006a,b; Yuzefovych et al., 2013; Vartanian et al., 2017; Komakula et al., 2018). Further, most of the significant changes related to mitochondrial function and content were observed in WAT, rather than in other tissues of *A^y^/a^Tg^* mice. These data are consistent with our prior observations of an important role for WAT remodeling in mediating the metabolic phenotypes of Ogg1Tg mice (Komakula et al., 2018). We thus propose that *hOGG1* expression increases mitochondrial content in *A^y^/a^Tg^* mice, particularly in WAT, resulting in elevated energy expenditure and attenuation of body weight, relative to *A^y^/a* littermates.

### Maternal Transmission of the *hOGG1* Transgene Is Necessary for Obesity Resistance

**FIGURE 4:**
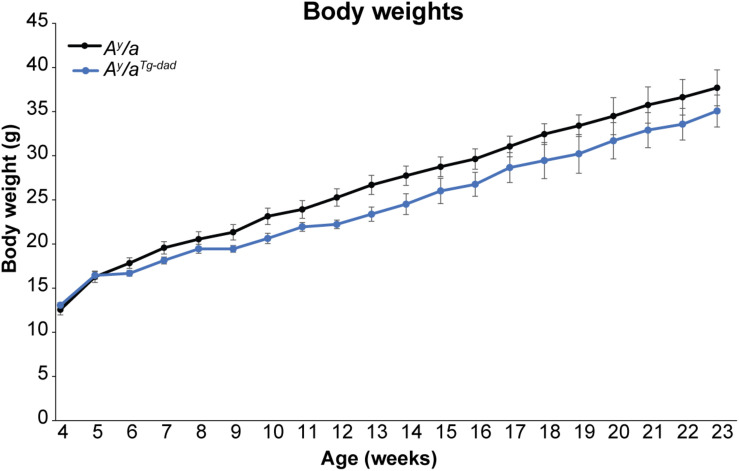
Body weights following paternal transmission of *hOGG1*. Body weights were measured at weekly intervals in female mice. Averages ± SEM. *p < 0.05 vs. *A^*y*^/a.*

Breeding of *A^y^/a* mice is inherently challenging, as the *A^y^/a* mutation reduces fecundity and is embryonically lethal in offspring of homozygous mutants. Thus, animals used in the studies above were derived from parents where one parent transmitted the *A^y^/a* mutation, with the female parent carrying the *hOGG1* transgene. However, during colony expansion, we generated a limited number of offspring where the *hOGG1* transgene was transmitted solely by the male parent (*A^y^/a^Tg^*^–^*^*dad*^*), since the female parent did not carry the *hOGG1* transgene. Intriguingly, we noticed that offspring from this mating were not protected from obesity, relative to *A^y^/a* controls. Thus, while offspring of female *A^y^/a^Tg^* mice were protected from agouti-induced obesity, offspring of male *A^y^/a^Tg^* mice were not (Figure 4). Regrettably, these mice were euthanized at 24 weeks, hence longer term body weight curves were not generated for this colony.

**FIGURE 5:**
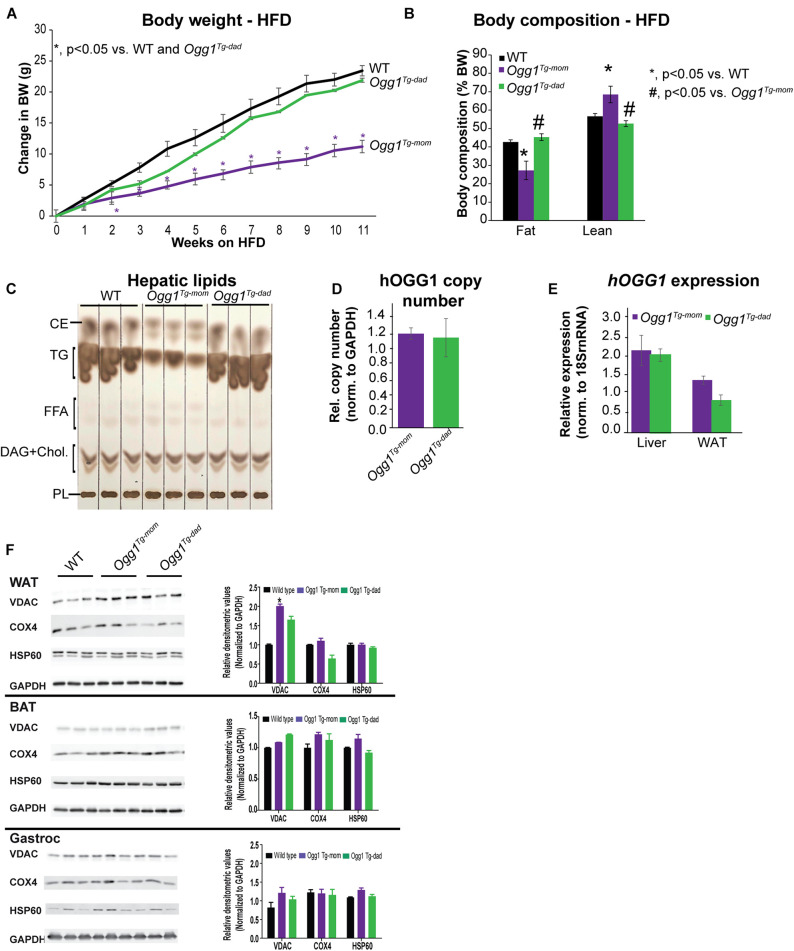
Maternal vs. paternal transmission of *hOGG1* influences metabolic phenotype and mitochondrial content of WAT. **(A)** *Ogg1*^*Tg*–^ *^*mom*^* or *Ogg1*^*Tg*–^ *^*dad*^* animals were placed on high-fat diets (HFDs) at 8 weeks of age, and body weights were measured weekly. **(B)** Body composition was measured at the end of 11 weeks of HFD feeding. **(C)** Hepatic lipids were extracted and separated by thin-layer chromatography. **(D)** Relative hOGG1 copy number and **(E)** *hOGG1* gene expression were measured by qPCR. **(F)** Tissue expression of mitochondrial markers was measured by immunoblotting. CE, cholesterol esters; DAG, diacylglycerols; FFA, free fatty acids; PL, phospholipids; TG, triglycerides.

Since *A^y^/a^Tg^* females were lighter than *A^y^/a* animals at breeding age (8 weeks), we sought to determine if the differences in offspring phenotype were simply a function of maternal body weight during pregnancy. We therefore established breeding colonies in our non-*A^y^/a* colony, such that either the female or male parent was *hOGG1* transgenic (*Ogg1*^*Tg*–^*^*mom*^* or*Ogg1^*Tg*^*^–^*^*dad*,^* respectively). Under chow-fed conditions, WT and *Ogg1*^*Tg*^ mice do not differ in body weights or litter sizes. WT and *Ogg1*^*Tg*^ offspring from all breeding pairs were placed on a 12-week HFD, starting at 8 weeks of age. Mice with maternal transmission of the *hOGG1* transgene (*Ogg1*^*Tg*–^*^*mom*^*) were significantly protected from HFD-induced body weight gain, relative to WT littermates, as we have previously reported (Figure 5A). However, mice that received the transgene solely from the male parent (*Ogg1*^*Tg*–^*^*dad*^*) had body weights that were indistinguishable from WT littermates when fed a HFD (Figure 5A). Body composition analyses revealed that fat mass was significantly reduced in *Ogg1*^*Tg*–^*^*mom*^* mice, relative to WT counterparts (Figure 5B). Additionally, lean mass was increased in *Ogg1*^*Tg*–^*^*mom*^* mice, relative to WT controls. In contrast, fat and lean masses in *Ogg1*^*Tg*–^*^*dad*^* mice were indistinguishable from WT controls and significantly higher than *Ogg1*^*Tg*–^*^*mom*^* animals (Figure 5B). Hepatic lipid accumulation is a distinct risk factor for further metabolic dysfunction, and we have previously shown that while OGG1 deficiency increases hepatic lipid storage, OGG1-overexpression significantly reduces hepatic lipids (Sampath et al., 2012b; Komakula et al., 2018). Here we discovered that while stored hepatic lipids such as triglycerides (TG) and cholesterol esters (CE) were significantly reduced in *Ogg1*^*Tg*–^*^*mom*^* mice, no such reduction was apparent in *Ogg1*^*Tg*–^*^*dad*^* livers (Figure 5C). Thus, transgene inheritance from the female parent was both required and sufficient to confer protection against HFD-induced weight gain, whole body adiposity, and hepatic lipid accumulation.

### Increased Mitochondrial Content in WAT Requires Maternal Transmission of the *hOGG1* Transgene

Inheritance of the *hOGG1* transgene from the male or female parent did not alter the relative transgene copy number nor gene expression of *hOGG1* (Figures 5D,E). This led us to conclude that differences in obesity resistance were not a function of unexpected differences in copy number or *hOgg1* expression levels in the offspring. We have previously shown that in the context of HFD-induced obesity, changes in mitochondrial content and function in WAT may mediate obesity resistance in *Ogg1*^*Tg*^ mice. Consistent with these previous reports, we discovered that *Ogg1*^*Tg*–^*^*mom*^* mice had increased mitochondrial content, reflected in significantly increased content of VDAC in WAT. However, *Ogg1*^*Tg*–^*^*dad*^* animals did not have a similar increase in mitochondrial content, consistent with the lack of obesity resistance in these mice (Figure 5F). Similar increases in mitochondrial content were not observed in BAT or gastroc of *Ogg1*^*Tg*^ mice (Figure 5F). These findings are consistent with our previous reports (Komakula et al., 2018) and suggest that differences in metabolic phenotypes in *Ogg1*^*Tg*^ mice may stem from alterations in WAT mitochondrial content and function. Overall, our results indicate that maternal transmission of the *hOGG1* transgene confers resistance not only to genetically-induced obesity (Figure 4), but also to HFD-induced obesity.

## Discussion

We demonstrate for the first time that enhanced expression of the human *OGG1* gene confers protection against genetically-induced obesity. The Agouti yellow mouse obesity syndrome is a result of dominant mutations at the Agouti locus (Moussa and Claycombe, 1999). Ectopic expression of Agouti in multiple tissues in these mice results in a yellow coat color, chronic hyperphagia, obesity, increased linear growth, leptin and insulin resistance, and hyperglycemia. Mechanisms mediating hyperphagia and obesity in this model involve antagonism of the melanocortin receptor, and similar phenotypes have been described in mice overexpressing Agouti-Related protein (AGRP), a potent antagonist of the melanocortin receptors-3 and -4 (Moussa and Claycombe, 1999; Small et al., 2001). Human obesity is also frequently associated with resistance to both leptin and insulin, and genome-wide association studies have implicated mutations near the melanocortin receptor-4 in the development of obesity and insulin resistance (Chambers et al., 2008; Loos et al., 2008). Thus, our results indicating a role for OGG1 in attenuating body weights in the *A^y^/a* obese model (Figure 1) are particularly relevant from a translational standpoint. As *hOGG1* expression lowered body weight without lowering food intake (Figure 1), body weight reduction in *A^y^/a^Tg^* mice likely results from alterations in peripheral tissues. Consistently, we observed increases in whole body energy expenditure (Figure 2) and mitochondrial content in several metabolically important tissues, particularly in WAT of *A^y^/a^Tg^* mice (Figure 3).

An intriguing discovery of these studies is that of obesity resistance being determined by the maternal OGG1 genotype. Male *Ogg1*^*Tg*^ parents were unable to transmit obesity resistance to their *Ogg1*^*Tg*^ offspring. Female *Ogg1*^*Tg*^ mice, conversely, could transmit this metabolically beneficial phenotype to both male and female (not shown) offspring. These data are strongly suggestive of the mitochondrial genome, which is inherited solely from the female parent, as being a critical determinant of the obesity resistance phenotype in *Ogg1*^*Tg*^ mice.

Studies examining the developmental origins of disease have established a strong link between maternal obesity and adverse metabolic outcomes in offspring (Das et al., 2021). For instance, in both human and rodent studies, maternal obesity is associated not only with increased risk of pregnancy complications such as gestational diabetes, but also with adverse outcomes in the offspring, including increased risk of obesity and diabetes. The mechanisms underlying these effects are not completely known but likely involve hormonal regulation, epigenetic changes, oxidative stress in the uterine environment, and dysregulated gut microbiota, among other factors (Saben et al., 2016; Das et al., 2021). While these links between maternal obesity and fetal outcomes have been extensively studied in animal models, relatively few studies have examined a link between maternal obesity resistance and metabolic outcomes in the offspring. Our studies indicate that maternal, but not paternal, OGG1 genotype influences tissue mitochondrial content and energy balance in both male and female *Ogg1*^*Tg*^ offspring. These findings clearly implicate a role for mitochondrial quality in determining obesity resistance phenotypes in offspring. They also raise intriguing questions about interactions between the intrauterine environment, which is influenced by maternal genotype, and offspring genotype. Prior studies have reported roles for increased maternal oxidative stress and DNA damage in impacting comparable stress in the fetus and offspring (Luo et al., 2006; Simmons, 2006; Raicević et al., 2010; Rodríguez-Rodríguez et al., 2018). Further, epigenetic modifications during development have been shown to be influenced by an adverse fetal environment and to, in turn, impair metabolic outcomes in adult offspring (Seki et al., 2012; Houde et al., 2013; Ruchat et al., 2013; Marchlewicz et al., 2016; Zhu et al., 2019). In this regard, 8-oxoG itself may serve as an epigenetic mark, thereby altering promoter transcription rates *via* the recruitment of OGG1 to sites of oxidation (Perillo et al., 2008; Ruchko et al., 2009; Zarakowska et al., 2014; Allgayer et al., 2016; Fleming et al., 2017a, b; Hao et al., 2020). In addition to being recruited to 8-oxoG sites, OGG1 activity influences DNA methylation, as oxidized guanines in CpG sequences are resistant to the action of DNA methyltransferases (Weitzman et al., 1994; Valinluck et al., 2004; Maltseva et al., 2009; Ba and Boldogh, 2018). Thus, oxidative stress and OGG1 activity are both critical determinants of epigenetic programming, although their roles in the uterine environment have not been investigated. We observed that protection from DIO required both the female parent and offspring to carry the hOGG1 transgene. Thus, it is possible that reduced uterine oxidative stress in an *Ogg1*^*Tg*^ female may result in altered metabolic programming in *Ogg1*^*Tg*^ offspring. The mechanisms mediating these interactions between maternal or *in utero* genotype with offspring genotype require further investigation.

These studies also suggested that among markers of mitochondrial content, which can directly influence energy expenditure, increased mitochondrial content in WAT was consistent with obesity resistance in *Ogg1*^*Tg*–^*^*mom*^* but not *Ogg1*^*Tg*–^*^*dad*^* mice (Figure 5F). These data further support our working hypothesis that the improved metabolic phenotype in *Ogg1*^*Tg*^ mice is a consequence of increased mitochondrial content and improved function in WAT (Komakula et al., 2018). In further support of a role for OGG1 in modulating adipocyte behavior, we recently showed that OGG1 genotype corresponds with adipocyte differentiation capacity (Komakula et al., 2021). Preadipocytes lacking OGG1 differentiated faster and accumulated more lipids than WT cells, while *hOGG1* expression significantly blunted adipocyte differentiation and lipid accretion. These phenotypes in isolated preadipocytes and 3T3 cells correspond with obesity predisposition or resistance in *Ogg1*^–^*^/^*^–^ and *Ogg1*^*Tg*^ animals, respectively (Komakula et al., 2021). They also indicate an important cell-intrinsic role for OGG1 in the adipocyte, as supported by our current studies.

In summary, we show here for the first time that *hOGG1* expression is protective against obesity resulting not only from HFD consumption, but also from genetically-induced obesity. In both models, *hOGG1* expression alters tissue mitochondrial content, particularly in WAT, and enhances overall energy expenditure. Importantly, maternal transmission of the transgene is both necessary and sufficient to confer resistance to obesity. These data have important implications to our understanding of the etiology of obesity and the role that DNA damage and repair may play in the process. They also establish an important role for interactions between the *in utero* environment, shaped by maternal genotype, with offspring genotype in impacting developmental programming and influencing metabolic outcomes in adult animals.

## Data Availability Statement

The original contributions presented in the study are included in the article/supplementary material, further inquiries can be directed to the corresponding author.

## Ethics Statement

The animal study was reviewed and approved by Animal Care and Use Committee of Rutgers University, New Brunswick, NJ, United States.

## Author Contributions

NB, RL, and HS contributed to conceptualization and design of the study. NB, PS, HY, SK, VV, and HS carried out experiments and analyzed data. NB, PS, and HS prepared figures and wrote the first draft of the manuscript. AD, RL, and HS edited the manuscript and obtained funding. All authors have read and approved the submitted version.

## Conflict of Interest

The authors declare that the research was conducted in the absence of any commercial or financial relationships that could be construed as a potential conflict of interest.

## Publisher’s Note

All claims expressed in this article are solely those of the authors and do not necessarily represent those of their affiliated organizations, or those of the publisher, the editors and the reviewers. Any product that may be evaluated in this article, or claim that may be made by its manufacturer, is not guaranteed or endorsed by the publisher.
